# Nutrition in Early Life and Its Impact Through the Life Course

**DOI:** 10.3390/nu17040632

**Published:** 2025-02-10

**Authors:** Haoran Ren, Yubo Zhou, Jianmeng Liu

**Affiliations:** 1 Institute of Reproductive and Child Health/National Health Commission Key Laboratory of Reproductive Health, School of Public Health, Peking University, 38 Xueyuan Rd., Beijing 100191, China; 2311110183@pku.edu.cn (H.R.);; 2 Department of Epidemiology and Biostatistics, School of Public Health, Peking University, 38 Xueyuan Rd., Beijing 100191, China

The term “early life” refers to the period spanning from the fetal stage to the age of two years after birth, encompassing a total duration of approximately 1000 days [1,2]. The “first 1000 days of life” is the most critical period for the rapid development and maturation of organs and systems [2], and thus necessitates a diverse array of nutrients derived from maternal placental transfusion during pregnancy, through breastfeeding during infancy, and from complementary foods thereafter [3].

Nutrition in early life may program the growth trajectory and immune system of childhood and lifelong health. In the 1980s, David Barker first proposed that lower birth weight was associated with increased risk of ischemic heart disease mortality in adulthood [4,5]. In 1995, Barker formally proposed “the fetal origins hypothesis”, which stated that mid-late gestational undernutrition drives developmental changes that increase lifelong cardiovascular risk [6], which evolved into the Developmental Origins of Health and Disease (DOHaD) hypothesis [7]. The hypothesis stimulated numerous studies that reported the impact of early life nutrition, either inadequate or excessive, on non-communicable chronic diseases. In addition to birth cohort studies from Finland [8,9], evidence from the Netherlands [10,11] and China [12,13] consistently demonstrates that offspring exposed to nutritional deprivation during the fetal period or after birth were more prone to metabolic syndrome, type 2 diabetes, or cardiovascular diseases in middle and late adulthood. On the contrary, excessive nutrition in early life, such as maternal prepregnancy obesity and excessive gestational weight gain, are also risk factors for cardiovascular disease in offspring during childhood and adulthood [14,15].

Neurodevelopment and lifelong mental health may also be programmed by nutritional status in early life. Previous studies showed that neonates with blood glucose levels of ≤30 mg/dL had lower full-scale intelligence quotient (IQ) scores and higher risk of abnormal fine motor function and visual–motor integration [16,17]. Although macronutrient sufficiency is necessary for optimal brain development, most supplementation trials have been focused on key micronutrients, such as folate, docosahexaenoic acid (DHA), iron, and zinc. It is widely agreed that supplementation with folic acid peri- and during early pregnancy is critical to preventing neural tube defects [18]. Regarding DHA supplementation, the most beneficial effects were reported for preterm newborns who were supplemented with DHA, who displayed improved white matter maturation and higher full-scale IQ scores in childhood [19,20]. Iron and zinc are widely recognized trace elements that significantly influence neurodevelopment. The single supplementation of iron [21] and zinc [22] in infants would improve their motor and neurocognitive development in later life. Moreover, the co-supplementation of iron and zinc has significantly enhanced beneficial effects compared to single supplementation [23].

Multiple nutrient imbalances in early life tend to occur simultaneously, mainly due to suboptimal dietary patterns such as the poor diet quality of mothers and inappropriate feeding practices for infants and toddlers [24]. The inadequate maternal intake of either animal-source foods or plant-based foods would mean that the requirements of offspring for essential nutrients are not met. A better diet quality during pregnancy and lactation is associated with greater white matter development in the offspring’s brain [25], higher intelligence, and better executive functioning skills in childhood [26,27]. For infants in the first 6 months of life, exclusively breastfeeding as recommend by WHO [28] significantly reduced their morbidity and mortality [29] and improved neurobehavioral development [30]. For infants aged 6 months to 2 years, sufficient quantities and adequate quality of complementary foods, together with their timely introduction at 6 months of age, are critical not only for the immediate development of children but also for their health over a lifetime [31].

In summary, the period of the “first 1000 days of life” could be regarded as a time of tremendous opportunity as well as a time of great vulnerability for later health. As public health nutrition has continued to develop, our understanding of the impact of early-life nutrition on long-term health outcomes has deepened, but there are still several areas that should be paid more attention. The first is that, although the first 1000 days of life were usually viewed as a continuum period, the most sensitive critical window to promote health and development should be explored for specific key nutrients. For example, from 1 month before conception to the first 2–3 months of pregnancy is the critical period for maternal folate status to guarantee the neural tube formation. The second is that more studies are needed to explore the effects of neglected nutrients, such as choline, in both developed and developing countries. Finally, understanding the interplay of multiple nutrient imbalances is essential to provide specific implications for prioritizing public policies for pregnant and breastfeeding women, infants, and toddlers.
